# First Clinical Results of Hyperopic Eyes Treated with a New Ablative Solid-State Laser

**DOI:** 10.3390/medicina61030395

**Published:** 2025-02-25

**Authors:** Bojan Pajic, Zeljka Cvejic, Anna Schroeter, Valentin Pajic, Anthia Papazoglou, Brigitte Pajic-Eggspuehler

**Affiliations:** 1 Eye Clinic ORASIS, Swiss Eye Research Foundation, 5734 Reinach, Switzerland; 2 Department of Physics, Faculty of Sciences, University of Novi Sad, Trg Dositeja Obradovica 4, 21000 Novi Sad, Serbia; 3 Division of Ophthalmology, Department of Clinical Neurosciences, Geneva University Hospitals, 1205 Geneva, Switzerland; 4 Faculty of Medicine, University of Geneva, 1205 Geneva, Switzerland; 5 Faculty of Medicine of the Military Medical Academy, University of Defense, 11000 Belgrade, Serbia; 6 Department of Mechanical and Process Engineering, ETH Zuerich, Raemistrasse 101, 8092 Zuerich, Switzerland; 7 Department of Ophthalmology, Cantonal Hospital of Aarau, 5001 Aarau, Switzerland; 8 Faculty of Medicine, University of Zurich, 8006 Zurich, Switzerland

**Keywords:** solid-state laser, ablation laser, refractive surgery, cornea surgery

## Abstract

Investigation and evaluation of the first clinical results of the new ablative solid-state laser (AQUARIUZ) regarding clinical outcome, inclusively higher-order aberration and safety. In this case report, three hyperopic patients with six eyes were treated with the new ablative solid-state laser (AQUARIUZ). The LASIK incisions are cut with the Ziemer LDV Z8. All patients were followed for 6 months postoperatively. The treated hyperopia ranged from +0.5 D to +2.75 D. Emmetropia of 0 D was found in four eyes after 6 months. In two eyes there was a slight myopia of −0.25 D each, which is also considered emmetropia according to the definition. The aspherically optimized profile of the ablative solid-state laser did not induce a higher-order aberration or spherical aberration in any eye. No eye lost CDVA or UDVA lines after the follow-up period. The safety index was 1 in five eyes and 1.25 in one eye. The findings demonstrate a high level of precision and treatment safety with the new ablative solid-state laser.

## 1. Introduction

The excimer laser has proven to be a safe and efficient technology in the correction of refractive errors [1,2]. Almost all excimer lasers used in ophthalmology operate at a wavelength of 193 nm, primarily because this wavelength, which is in the deep ultraviolet (UV) spectrum, is highly effective for photoablation [3,4]. However, there were also excimer lasers operating at a wavelength of 223 nm, which ultimately did not become established [5]. Photoablation is the process of precisely removing submicron-thick layers of corneal tissue without causing significant heat damage to surrounding tissues, making it ideal for refractive surgery applications [3]. Corneal absorption is relatively weak between 266 nm and 248 nm, which makes these wavelengths less effective for precise ablation without causing thermal effects [6,7]. Absorption increases significantly between 248 nm and 223 nm, indicating the corneal tissue’s capacity to absorb UV light and enable ablation down to submicron precision [3,7]. From 223 nm to 193 nm, absorption in corneal tissue increases further, making this range optimal for precise corneal reshaping applications with minimal collateral damage [3,4,8]. This property is crucial for maintaining the biomechanical integrity of the cornea during and after surgery [3,4,7,9]. Consequently, treatment using photoablation in this wavelength range results in high accuracy without damaging the adjacent corneal tissue. Experimental studies have shown that ablative solid-state lasers at a wavelength of 213 nm do not cause thermal damage to the corneal tissue after photorefractive keratectomy (PRK) and are therefore safe lasers [10,11].

Ablative solid-state lasers operating in the 205–215 nm wavelength range offer distinct advantages over excimer lasers in ophthalmology, particularly in refractive and therapeutic corneal surgeries. The advantages, such as reduced dependence on corneal hydration state reduced sensitivity and absorption variability, are primarily related to their interaction with the corneal tissue [12,13,14,15]. Excimer lasers (193 nm) are highly dependent on the corneal hydration level because the absorption in water increases drastically for wavelengths shorter than 200 nm [16]. Variability in hydration may lead to inconsistent ablation rates, affecting precision and predictability [12,13,14,15,17]. Ablation with a solid-state laser in the range of (205–215 nm), on the other hand, is less influenced by water absorption, providing greater consistency in tissue removal regardless of hydration variations [17,18,19,20,21]. This feature enhances surgical reliability, particularly in challenging clinical conditions or when treating patients with variable corneal hydration states [13]. At 205–215 nm, the laser beam is less affected by tissue-specific absorption variations compared to 193 nm excimer lasers because more UV light is absorbed in the collagen and less in the water of the cornea. This stability beneficially adds to more uniform ablation profiles, improving the predictability of surgical outcomes and reducing the risk of irregularities in corneal reshaping.

Solid-state lasers, without the need for toxic gases, simplify installation and reduce long-term operational expenses. These systems are more compact, easier to maintain, and more durable, making them suitable for a wider range of clinical settings, including mobile or low-resource environments [22]. Other potential advantages of an ablative solid-state laser over an excimer laser are lower power consumption as well as significantly lower ambient noise levels.

We have already shown that the ablative solid-state laser (AQUARIUZ) has excellent results for the correction of myopia [21]. In this paper, we will present the results of the first hyperopia treatments focusing on distance visual acuity, safety index, refraction, and higher-order aberrations in a case report.

## 2. Materials and Methods

We conducted a retrospective observational case series of 3 patients with 6 eyes. The procedures were carried out in accordance with the Declaration of Helsinki; in particular, all legal provisions were applied. Written informed consent was obtained from all patients. The procedures were performed in 2023 by the same surgeon (B.P.).

A detailed ophthalmological status was obtained in all patients, in particular manifest refraction, uncorrected distance visual acuity (UDVA), corrected distance visual acuity (CDVA), cycloplegic refraction, biomicroscopic findings of the anterior segment, dilated retinal examination, and eye pressure measurement. Using the Galilei system (GALILEI G2, Ziemer Ophthalmic Systems, Switzerland), corneal topographical data were collected, and aberrations were measured using the iTrace device. The wavefront measurement was performed in all eyes under scotopic conditions with a measurement zone of 6 mm. Corneal OCT (Topcon) was performed on all patients preoperatively and at each postoperative time point. The Femto-LASIK criteria were used for the surgical indication.

The AQUARIUZ UV ablative solid-state laser (Ziemer Ophthalmic Systems AG, Port, Switzerland) was used for refractive surgery. The FEMTO LDV Z8 femtosecond laser (Ziemer Ophthalmic Systems AG, Port, Switzerland) was applied to create a LASIK flap. The hinge was set superiorly. A flap diameter of 9.5 mm with a cutting depth of 110 µm was aimed for in all eyes. The AQUARIUZ creates nanosecond UV pulses in the wavelength range of 205–215 nm generated by frequency conversion of an infrared (IR) seed laser. The laser spot diameter was set to 0.6 mm. The laser spot was centered on the 1st Purkinje reflex. For all eyes, an optical zone of 6.5 mm was treated with a total ablation zone of 9.5 mm. The pulse repetition rate was 500 Hz. The system was integrated with a 6-dimensional eye-tracking system (including XY, gaze, and Z tracking). An optimized aspheric profile was used for the ablation profile.

**Figure 1:**
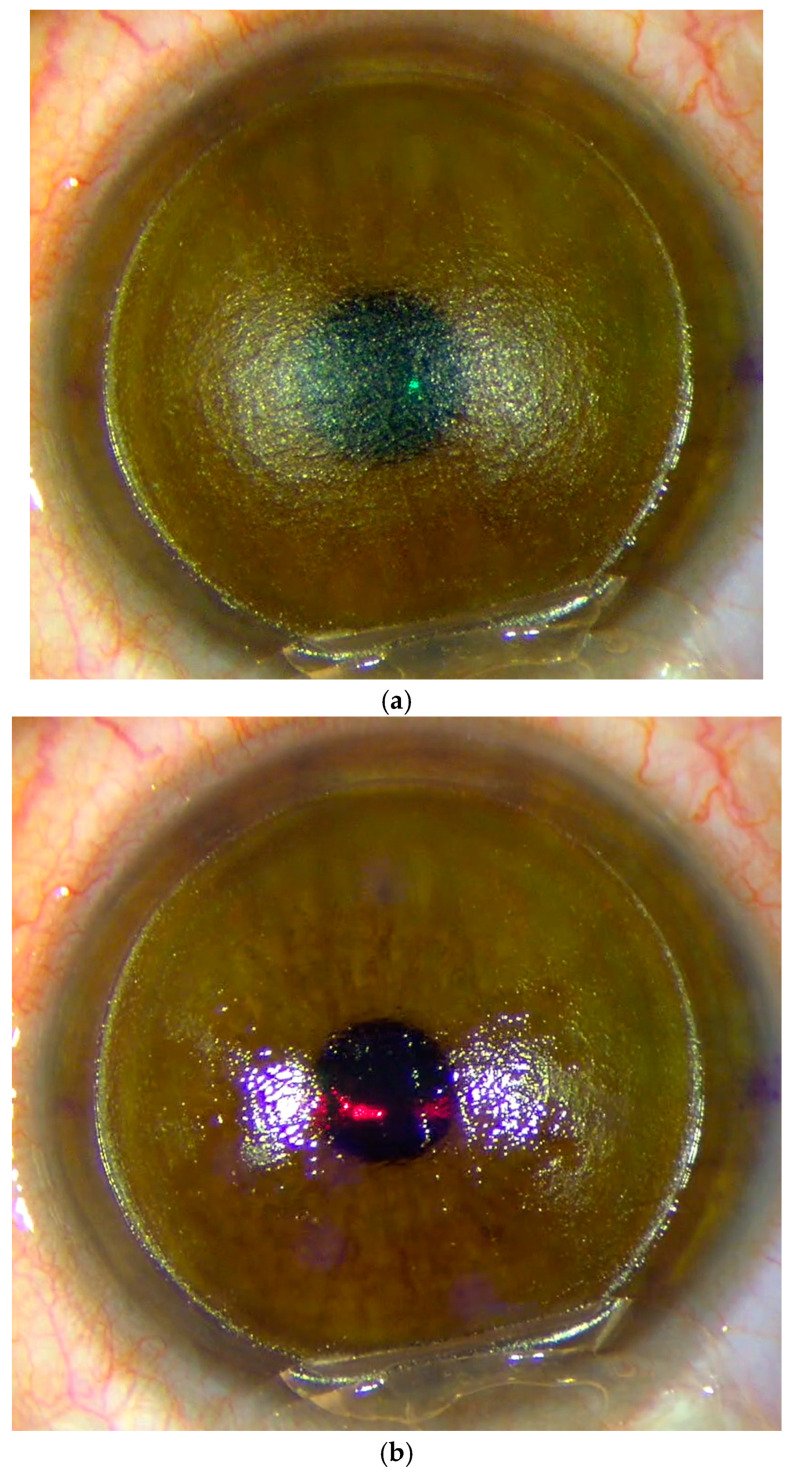

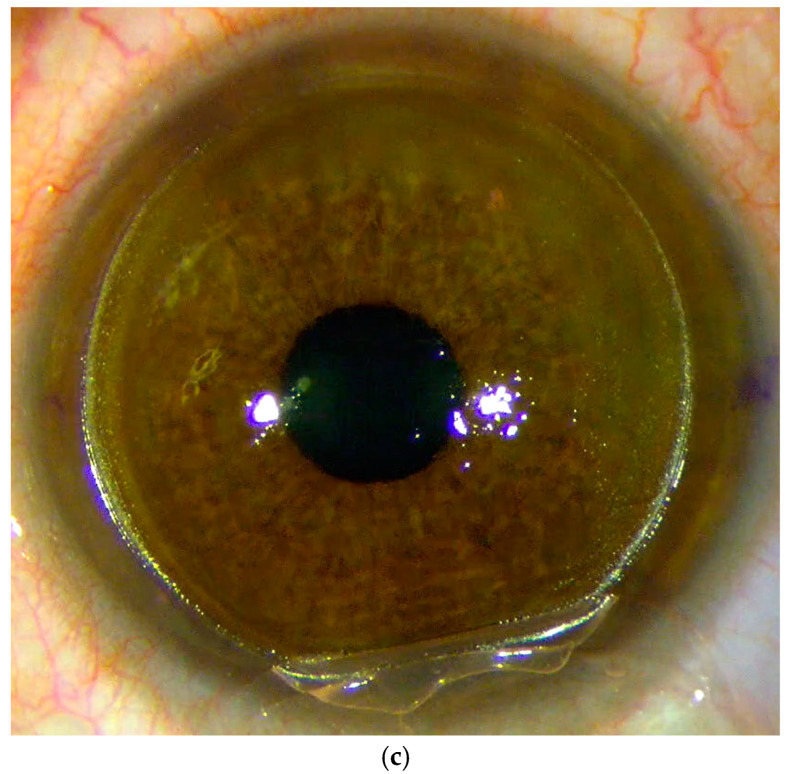
(**a**) Condition before treatment with ablative solid-state laser. (**b**) At the beginning of the application of the ablative solid-state laser, you can see how the liquid was pushed out of the corneal stroma. (**c**) In the process of application and at the end, it can be observed how the corneal bed was completely covered with fluid.

Due to the different laser wavelengths, ablative solid-state lasers show different treatment dynamics during application than excimer lasers. Figure 1a shows the condition after the flap incision with a dry stromal bed before laser application. At the beginning of the laser procedure, Figure 1b shows how fluid was pushed out of the stromal bed and how the stromal surface becomes completely covered by fluid as the laser was applied, as shown in Figure 1c. In contrast to the excimer laser, the ablative solid-state laser was not absorbed by the fluid.

Tobradex (Alcon Laboratories, Inc., Fort Worth, TX, USA) was applied to all patients three times a day for one week. At the same time and for four weeks, topical hyaluronic acid 0.15% was also applied three times a day.

## 3. Results

A total of six eyes from three patients were treated. A total of five of the six eyes were hyperopic astigmatisms, with astigmatism power of 0.25 to 1.25 D. The remaining eye was hyperopic. The mean patient age was 49.7 ± 16.2 years. In patient 1, the preoperative corrected distance visual acuity (CDVA) was OD 1.0, OS 1.0; in patient 2, OD 1.0 and OS 1.0; and in patient 3, OD 1.0 and OS 0.8 (Table 1).

The preoperative manifest refraction spherical equivalent (MRSE) was OD +0.38 D and OS +0.88 D in patient 1, OD +0.63 D and OS +0.38 D in patient 2, and OD +1.25 D and OS +2.75 D in patient 3. The target refraction was plano in all patient eyes. The uncorrected preoperative distance visual acuity (UDVA) was OD 0.8, OS 0.8 in patient 1, OD 0.5, OS 0.63 in patient 2, and OD 0.5, OS 0.32 in patient 3 (Table 2).

**Figure 2:**
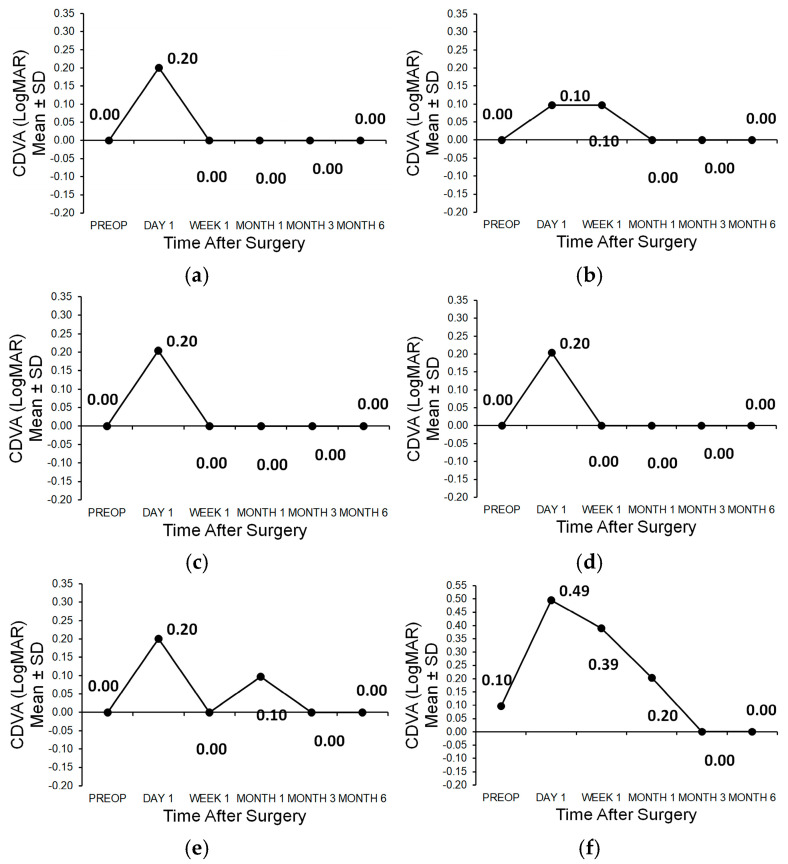
(**a**) Corrected distance visual acuity of patient 1 OD; (**b**) Corrected distance visual acuity of patient 1 OS; (**c**) Corrected distance visual acuity of patient 2 OD; (**d**) Corrected distance visual acuity of patient 2 OS; (**e**) Corrected distance visual acuity of patient 3 OD; (**f**) Corrected distance visual acuity of patient 3 OS.

In patient 1, the development of the corrected postoperative distance visual acuity (CDVA) is shown in (Figure 2a,b). The safety index was 1 after 6 months in both the right and left eyes. In patient 2, the follow-up of the corrected postoperative distance visual acuity (CDVA) is shown in (Figure 2c,d). The safety index after 6 months was 1 in both the right and left eyes. In patient 3, the development of the corrected postoperative distance visual acuity (CDVA) is shown in (Figure 2e,f). After 6 months, the safety index was 1 in the right eye and 1.25 in the left eye (Table 1).

**Figure 3:**
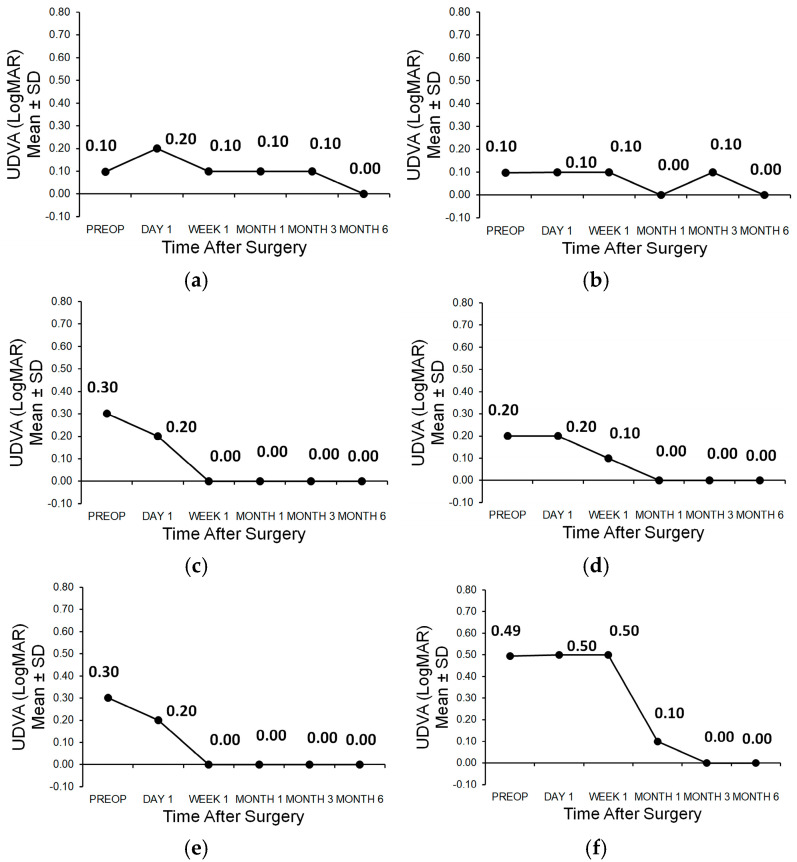
(**a**) Uncorrected distance visual acuity of patient 1 OD; (**b**) Uncorrected distance visual acuity of patient 1 OS; (**c**) Uncorrected distance visual acuity of patient 2 OD; (**d**) Uncorrected distance visual acuity of patient 2 OS; (**e**) Uncorrected distance visual acuity of patient 3 OD; (**f**) Uncorrected distance visual acuity of patient 3 OS.

In patient 1, the follow-up of the uncorrected postoperative distance visual acuity (UDVA) is shown in (Figure 3a,b). In patient 2, the outcome of the uncorrected postoperative distance visual acuity (UDVA) is shown in (Figure 3c,d). In patient 3, the course of the uncorrected postoperative distance visual acuity (UDVA) is shown in (Figure 3e,f). In the overview table, the uncorrected postoperative distance visual acuity (UDVA) is presented numerically (Table 2).

**Figure 4:**
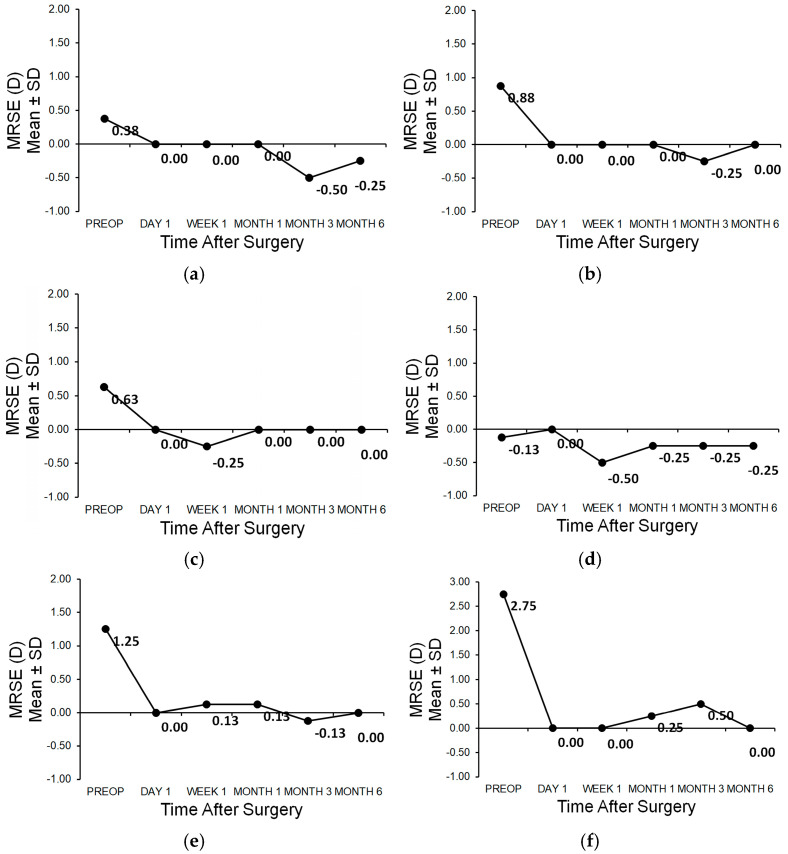
(**a**) Manifest refraction spherical equivalent of patient 1 OD; (**b**) Manifest refraction spherical equivalent of patient 1 OS; (**c**) Manifest refraction spherical equivalent of patient 2 OD; (**d**) Manifest refraction spherical equivalent of patient 2 OS; (**e**) Manifest refraction spherical equivalent of patient 3 OD; (**f**) Manifest refraction spherical equivalent of patient 3 OS.

The pattern of the manifest spherical refraction equivalent (MRSE) is shown in Figure 4a,b and Table 3 for patient 1. For patient 2, Figure 4c,d and Table 4 and for patient 3, Figure 4d,f and Table 5 show the follow-up of the manifest spherical refraction equivalent (MRSE).

Cycloplegic refraction (MRSE) was preoperative in patient 1 OD at +0.63 D and OS at +1.13 D, in patient 2 OD at +0.88 D and OS at +0.5, and in patient 3 OD at +1.63 and OS at +3.0 D.

**Figure 5:**
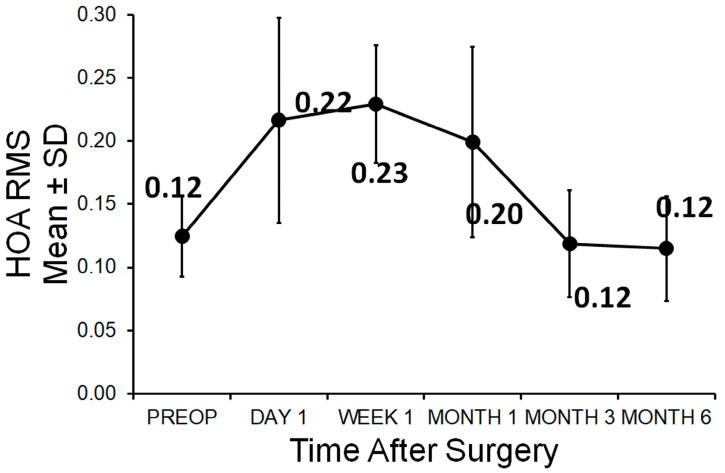
HOA RMS values during the course, preoperatively and at any time postoperatively.

In all eyes, the higher-order aberration root mean square (HOA) RMS and the spherical aberration (Z400) were measured in a zone of 6 mm. Preoperatively, the HOA RMS was 0.12 ± 0.03 µm. After one day postoperatively, there was an increase to 0.22 ± 0.08 µm. After 1 week, 1 month, and 3 months, the HOA RMS was 0.23 ± 0.05 µm, 0.20 ± 0.08 µm, and 0.12 ± 0.04 µm, respectively. After 6 months, the HOA RMS remained stable at 0.12 ± 0.04 µm (Figure 5).

**Figure 6:**
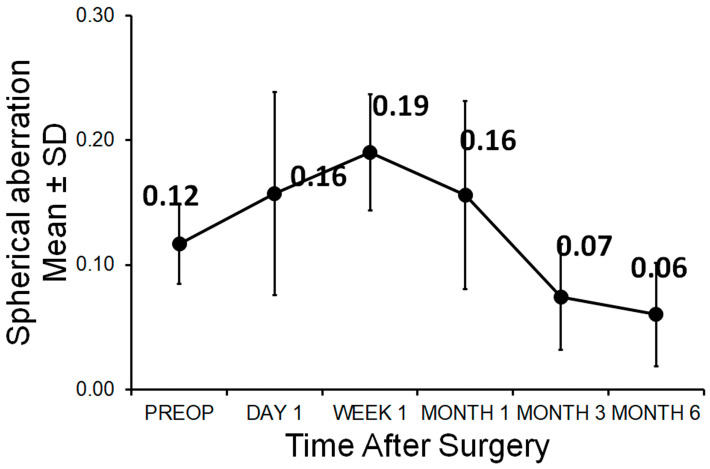
Spherical aberration Z400 values during the follow-up.

Preoperatively, the spherical aberration Z400 was 0.12 ± 0.07 µm. On the 1st postoperative day, the Z400 was measured at 0.16 ± 0.13 µm, after 1 week at 0.19 ± 0.05 µm and 1 month at 0.16 ± 0.09 µm. After 3 months and 6 months postoperatively, a Z400 of 0.07 ± 0.06 µm and 0.06 ± 0.05 µm could be observed (Figure 6).

## 4. Discussion

We have already demonstrated that the ablative solid-state laser showed excellent results in myopic patients. No nomograms were used for these cases [21]. In this case report, hyperopia was corrected with different degrees, i.e., eyes with a hyperopia of +0.5 D to +2.75 D were treated with the ablative solid-state laser. A slight overcorrection of −0.25 D was observed in two eyes after 6 months, whereas emmetropia was observed in four eyes. Excellent UDVA was observed in all eyes. The safety index was 1.0 and in one eye even 1.25 after 6 months postoperatively. Overall, the preoperative subjective refraction corresponded very well with the preoperative cycloplegic refraction values. In one eye, a slightly delayed increase in visual acuity was observed due to a transient dry eye.

The analysis of higher-order aberration (HOA) root mean square (RMS) highlights a significant advantage of the optimized aspheric profile utilized in the ablative solid-state laser: it does not induce any higher-order aberrations (HOAs), specifically spherical aberrations. This finding underscores the precision and efficacy of the aspheric profile in maintaining the optical quality of the treated cornea. Further validation of this outcome comes from an independent study on hyperopic patients treated with an ablative solid-state laser [23]. The study confirmed the absence of induced HOAs, supporting the conclusion that a laser system, with its optimized ablation profile, is potentially superior in preserving corneal integrity and visual quality post-treatment. Such results represent an advancement in refractive surgery, particularly for hyperopia correction, as they mitigate a common limitation associated with other laser platforms, which often results in postoperative visual disturbances due to induced HOAs. This is well in line with the observations made by several clinicians and research groups in terms of precision and patient outcomes in the field of refractive surgery [23,24,25,26]. The limitation of the work is the follow-up time of postoperative 6 months in a hyperopia procedure.

The very good clinical results, in particular the good safety and efficiency of the treatment using ablative solid-state lasers, can be compared with other research groups [24,25,26,27,28,29,30,31,32]. Solid-state lasers operate with significantly reduced acoustic noise compared to excimer lasers. This creates a quieter and more comfortable environment for both the patient and the surgical team, reducing procedural anxiety and enhancing the overall experience. Also, those lasers enable a wet ablation process, which contributes to less thermal stress on the cornea. By minimizing thermal stress during tissue removal, the risk of heat-induced effects on the surrounding corneal tissue is further reduced. The wet ablation process also facilitates more efficient removal of ablated material (debris) during surgery. This prevents residue buildup on the corneal surface, contributing to surgical precision and reducing postoperative inflammation or haze [21,26,27,28,29,30,31,32]. All the above-mentioned properties are beneficial for improved healing and recovery, especially for procedures such as hyperopic correction, where precision and tissue preservation are paramount.

## 5. Conclusions

The first results of hyperopia treatment with the new ablative solid-state laser show promising results, offering excellent visual outcomes, improved safety, and greater operational robustness compared to excimer laser systems. The treatment results of the new refractive solid-state laser technology are comparable to those of excimer lasers. These results pave the way for wider adoption of this technology in refractive surgery, potentially setting a new standard for hyperopia correction. Further long-term studies will help solidify its role in clinical practice and expand its applications to other refractive errors.

## Figures and Tables

**Table 1:** Pre- and postoperative CDVA (corrected distance visual acuity) in logMAR up to 6 months in the follow-up and safety indices.

Patient No.	Eye	CDVA Pre	CDVA 1 D	CDVA 1 W	CDVA 1 M	CDVA 3 M	CDVA 6 M	SI 6 M
1	OD	0	0.20	0	0	0	0	1
	OS	0	0.10	0.10	0	0	0	1
2	OD	0	0.20	0	0	0	0	1
	OS	0	0.20	0	0	0	0	1
3	OD	0	0.20	0	0.10	0	0	1
	OS	0.10	0.49	0.40	0.10	0	0	1.25

**Table 2:** Pre- and postoperative UDVA (uncorrected distance visual acuity) in logMAR up to 6 months in the follow-up and safety indices.

Patient No.	Eye	UDVA Pre	UDVA 1 D	UDVA 1 W	UDVA 1 M	UDVA 3 M	UDVA 6 M
1	OD	0.10	0.20	0.10	0.10	0.10	0.10
	OS	0.10	0.10	0.10	0	0.10	0
2	OD	0.30	0.20	0	0	0	0
	OS	0.20	0.20	0.1	0	0	0
3	OD	0.30	0.20	0	0	0	0
	OS	0.49	0.49	0.49	0.20	0	0

**Table 3:** Summary of manifest refraction spherical equivalent (MRSE) of patient 1.

Patient 1	Eye	Sph/D	Cyl/D	A/°	SEQ/D
PreOp	OD	+0.50	−0.25	130	0.38
	OS	+1.0	−0.25	80	0.88
1 Week	OD	0.00	0.00	0	0.00
	OS	+0.25	−0.50	75	0.00
1 Month	OD	0.00	0.00	0	0.00
	OS	+0.25	−0.50	70	0.00
3 Months	OD	−0.50	0.00	0	−0.50
	OS	−0.50	+0.50	40	−0.25
6 Months	OD	−0.25	0.00	0	−0.25
	OS	0.00	0.00	0	0.00

**Table 4:** Summary of manifest refraction spherical equivalent (MRSE) of patient 2.

Patient 2	Eye	Sph/D	Cyl/D	A/°	SEQ/D
PreOp	OD	+1.25	−1.25	125	0.63
	OS	+0.75	−0.75	105	0.38
1 Week	OD	−0.25	0.00	0	−0.25
	OS	−0.50	0.00	0	−0.50
1 Month	OD	0.00	0.00	0	0.00
	OS	−0.25	0.00	0	−0.25
3 Months	OD	0.00	0.00	0	0.00
	OS	−0.25	0.00	0	−0.25
6 Months	OD	0.00	0.00	0	0.00
	OS	−0.25	0.00	0	−0.25

**Table 5:** Summary of manifest refraction spherical equivalent (MRSE) of patient 3.

Patient 3	Eye	Sph/D	Cyl/D	A/°	SEQ/D
PreOp	OD	+1.50	−0.50	135	1.25
	OS	+2.75	0	0	2.75
1 Week	OD	+0.50	−0.75	175	0.13
	OS	0.00	0.00	0	0.00
1 Month	OD	+0.50	−0.75	175	0.13
	OS	+0.25	0.00	0	0.25
3 Months	OD	+0.25	−0.75	170	0.13
	OS	+0.50	0.00	0	0.50
6 Months	OD	0.00	0.00	0	0.00
	OS	0.00	0.00	0	0.00

## Data Availability

The data presented in this study are available upon request from the authors, in particular the datasets are archived in the clinics treated. The data are not publicly available as they contain information that could compromise the privacy of the participants.
